# Women in chemistry: Q&A with Dr Qi Hao

**DOI:** 10.1038/s42004-025-01430-4

**Published:** 2025-02-12

## Abstract

Dr Qi Hao is a Director, Protein Sciences, at Calico Life Sciences, USA.

Qi’s career began as a Ph.D. student at Tsinghua University, in the lab of Nieng Yan, where she studied signaling pathways of plant hormones and apoptosis. From there, she performed biochemical and biophysical studies for microtubule-associated protein complexes as a visiting scholar in Tarun Kapoor’s lab at Rockefeller University. She went on to complete her postdoc in the Gunter Blobel lab, where she used electron microscopy to study the transmembrane protein complex within the nuclear pore complex. Currently, she leads the Protein Sciences group at Calico Life Sciences supporting the company’s aging research and drug development programs.

**Figure:**
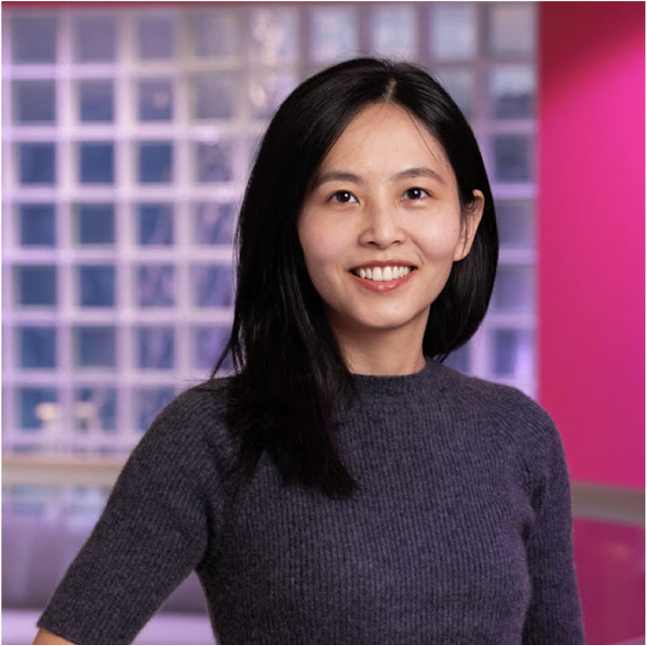
Calico Life Sciences

Why did you choose to be a scientist?

My fascination with the life sciences began at a young age, sparked by my father, who was a passionate biology teacher. This early interest led me to pursue scientific research in graduate school and beyond, drawn to the complexities and endless possibilities for discovery within the life sciences. I eventually became captivated by the challenges of aging-related research and drug development—a field that is as intricate and ambitious as it is intriguing. This passion ultimately brought me to Calico Life Sciences, where I’ve continued to pursue my scientific research career.

What scientific development are you currently most excited about?

I’m incredibly interested in the rapid development of new technologies which are accelerating research and drug development, particularly AI-based drug design. With my background in structural biology and biochemistry, I’m especially eager to embrace the interdisciplinary collaborations these advancements enable between research groups and computing teams. This is truly an exciting time to be at the forefront of these new technologies and it is thrilling to witness the daily progress in this field which has the potential to revolutionize drug development.

What direction do you think your research field should go in?

As mentioned, I’m incredibly enthusiastic about AI’s transformative impact on structural biology. The AI revolution extends far beyond structure prediction, with diverse machine learning models now driving various molecular design tasks and significantly elevating the drug development process. To further advance these AI models, the field needs to create more customized, high-throughput experimental approaches for generating robust training and validation datasets, an effort that requires interdisciplinary collaboration between computing, experimental, and engineering teams.

What aspects of your current work do you find most exciting or most rewarding?

Collaboration is one of the most rewarding aspects of being a scientist at an organization like Calico. Developing new technologies and drugs is always a team effort, and I deeply value the opportunity to learn from the many experts in our research and technology groups who bring diverse backgrounds to their work. While my own scientific training and current focus lie primarily in in vitro studies, I gain invaluable insights into in vivo work, preclinical, and clinical research by collaborating with my colleagues.

It is also very rewarding to know that the technologies and molecules I help develop might contribute to better solutions for easing the burden on our aging population and their families.

What do you most enjoy about being a scientist in your current position?

One of the most enjoyable aspects of my current role is the opportunity for continuous learning. The people at Calico represent a diverse range of expertise so I’m constantly gaining new knowledge from my peers, while also being involved in the drug development process and staying abreast of cutting-edge technologies.

Knowing that the research and technologies I contribute to have the potential to help people live longer and healthier lives also provides a sense of purpose that has become a significant part of what I enjoy about being a scientist.

What impact has your gender had on your career as a scientist?

In 2023, I was honored to be recognized as one of the ‘Next Generation Life Science Female Researchers’ by the San Francisco Business Times. This recognition further inspired me to support other women in science, particularly those early in their careers. Today, I actively mentor graduate students and postdoctoral researchers, offering guidance and advice as they navigate their career paths.

Are you or have you been supported by a mentor? What was the best advice you received?

Throughout my career, both in academia and industry, I’ve benefited immensely from supportive mentors. My PhD mentor, Professor Nieng Yan, was instrumental in my development. She not only provided rigorous technical training but also cultivated my critical thinking capabilities and my ability to multitask—skills that continue to serve me well in my career. Beyond scientific training, she has been a constant source of support for my career decisions. For example, when I was considering transitioning from academia to industry, she offered invaluable advice and helped me weigh the pros and cons of each path. Moreover, she is a dedicated advocate for up-and-coming female scientists, organizing forums and events to support our success. I also receive invaluable support from my industry mentor, Dan Eaton who is the Head of Development Technologies at Calico. His extensive industry experience proved essential in helping me make the transition from academia. He has coached me on establishing effective cross-organizational collaborations, prioritizing and executing tasks, and building a productive team.

‘Never give up’ was the most valuable advice I ever received and advice I continue to offer. Challenges and setbacks are inevitable, but they offer invaluable learning opportunities for growth and improvement.

*This interview was conducted by the editors of Communications Chemistry*.

